# NLR–FAR Index as a superior predictor of 30-day functional outcome after endovascular thrombectomy in acute ischemic stroke

**DOI:** 10.3389/fneur.2026.1703841

**Published:** 2026-02-25

**Authors:** Xuchen Meng, Weijie Zhong, Dingzhong Tang, Zixian Mei, Tanjun Deng, Xin Lv, Jiexi Xiao, Yueqi Zhu, Yi Li

**Affiliations:** 1 Neurosurgery Department, Shanghai Ninth People's Hospital Affiliated to Shanghai Jiao Tong University School of Medicine, Shanghai, China; 2 Neurology Department, Jinshan Branch of Shanghai Sixth People's Hospital, Shanghai, China; 3 Department of Radiology, Shanghai Sixth People's Hospital Affiliated to Shanghai Jiao Tong University School of Medicine, Shanghai, China

**Keywords:** acute ischemic stroke, biomarker, endovascular thrombectomy, prognosis factors, Systemic inflammatory

## Abstract

**Objective:**

To determine whether the neutrophil-to-lymphocyte ratio–fibrinogen-to-albumin ratio (NLR–FAR) Index predicts long-term prognosis in acute ischemic stroke patients undergoing endovascular thrombectomy (EVT).

**Introduction:**

Systemic inflammatory imbalance contributes to ischemic brain injury, but the combined effect of NLR and FAR on EVT outcomes has remained unclear.

**Methods:**

We retrospectively analyzed patients treated with EVT. A composite inflammatory-coagulation index, designated as the NLR–FAR Index, was defined as the product of NLR and FAR. The primary endpoint was 30-day functional outcome defined by the modified Rankin Scale (mRS), with poor outcome as mRS ≥3. Logistic regression and restricted cubic spline analyses were used to assess associations.

**Results:**

Of 254 patients, 121 (47.6%) had poor outcomes. Higher NLR, FAR, and especially NLR–FAR Index were significantly associated with poor prognosis. The NLR–FAR Index showed the strongest predictive effect (adjusted *OR* = 1.910, 95% CI: 1.079–3.384). Restrictive cubic spline analysis shows that when the NLR-FAR index exceeds the threshold of 0.62, the risk of adverse outcomes at 30 days begins to significantly increase.

**Conclusion:**

The NLR–FAR Index independently predicts functional outcomes after EVT and outperforms NLR or FAR alone. This accessible biomarker may aid early risk stratification and individualized management in acute ischemic stroke.

## Introduction

Acute ischemic stroke (AIS) remains a leading cause of mortality and long-term disability worldwide (1, 2). Although endovascular thrombectomy has substantially improved patient outcomes (3, 4), a considerable proportion of individuals continue to experience poor recovery. Consequently, the identification of reliable and easily accessible biomarkers for prognostication in AIS has become a key priority in clinical practice (5, 6).

Systemic inflammation plays a pivotal role in the pathophysiology of AIS, influencing both the extent of initial neuronal injury and the trajectory of subsequent recovery (7). The emerging concept of thrombo-inflammation refers to the deleterious interplay between immune activation and the coagulation cascade, now recognized as a central mechanism driving ischemic damage (8). Following arterial occlusion, a rapid and localized inflammatory response is triggered. At the site of occlusion, platelet activation promotes the release of chemokines such as CXCL4 (platelet factor 4) and CXCL7 (neutrophil-activating peptide 2), which recruit and activate neutrophils (9). In turn, neutrophils exacerbate injury through several mechanisms, including the release of neutrophil extracellular traps (NETs), which contribute directly to blood–brain barrier disruption, thrombosis, and impaired vascular repair (10, 11). This complex crosstalk between inflammatory and thrombotic processes highlights that the post-stroke environment is not solely inflammatory or prothrombotic, but represents a distinct thrombo-inflammatory state that critically influences clinical outcomes — even in patients who undergo successful endovascular thrombectomy (EVT) (12).

Given its central role in disease pathophysiology, readily accessible hematological indices that reflect systemic inflammation and coagulation have been extensively evaluated for their prognostic value. Among these, the neutrophil-to-lymphocyte ratio (NLR)—which integrates signals from the innate (neutrophils) and adaptive (lymphocytes) immune systems—has been the focus of considerable investigation. Elevated NLR values at admission have been consistently associated with greater stroke severity, larger infarct volumes, and poorer functional outcomes (13, 14). For example, in a retrospective study of 201 patients with acute ischemic stroke, Kim et al. (13) reported that a higher NLR was significantly associated with higher NIHSS scores (*p* = 0.011) and unfavorable outcomes. The prognostic value of NLR extends beyond stroke, having been robustly validated in patients with cancer (15).

Similarly, the fibrinogen-to-albumin ratio (FAR), representing the balance between a key procoagulant factor (fibrinogen) and a negative inflammatory biomarker (albumin), has demonstrated significant prognostic value in stroke. Higher FAR levels are linked to increased risks of stroke recurrence and unfavorable functional outcomes (16–18). A large cohort study of 809 patients with large artery atherosclerosis stroke by Wang et al. (16) found that after multivariable adjustment, a high FAR level was independently associated with an increased risk of stroke recurrence (hazard ratio, 2.57 [95% CI, 1.32–5.02]) and poor functional outcome (odds ratio, 3.30 [95% CI, 1.57–6.94]). Like NLR, FAR has shown prognostic relevance in diverse clinical contexts characterized by inflammation and coagulopathy, such as pancreatic cancer (19) and type 2 diabetes (20), reinforcing its biological plausibility as a composite indicator.

A critical synthesis of the literature reveals substantial limitations that hinder the adoption of single-parameter indices as reliable, standalone prognostic tools. First, their predictive performance exhibits marked heterogeneity across populations and study designs. For example, reported optimal cut-off values for the NLR vary considerably, and its prognostic utility appears to be influenced by stroke subtype and the timing of measurement (13, 14). Similar constraints are evident for the fibrinogen-to-albumin ratio (FAR), whose predictive accuracy may be confounded by variations in nutritional status and the presence of comorbidities (18, 20).

More fundamentally, each biomarker reflects only a partial aspect of the thrombo-inflammatory spectrum. NLR primarily indicates systemic immune cell activation but provides limited information on coagulation cascade activity or endothelial function—both critical in thrombus formation and stability (12). In contrast, FAR is sensitive to hypercoagulability and nutritional/inflammatory status but offers minimal insight into cellular immune responses, particularly the activation and balance of specific leukocyte subsets (17). Crucially, neither NLR nor FAR adequately captures the synergistic interaction between these pathways—the core feature of thrombo-inflammation (9, 10).

We hypothesize that a composite measure, the NLR–FAR Index, which integrates the neutrophil-to-lymphocyte ratio (NLR) and the fibrinogen-to-albumin ratio (FAR), can harness the complementary biological signals of its components to more comprehensively reflect the thrombo-inflammatory state. By concurrently capturing cellular inflammation through NLR and coagulation–nutritional disturbances through FAR, this novel index may enhance the prognostic accuracy for functional outcomes following endovascular therapy (EVT) and address the limitations associated with each individual marker.

## Methods

### Study design and population

A retrospective observational study was conducted, enrolling 254 patients diagnosed with acute ischemic stroke (AIS) who were admitted to the Department of Neurology at Shanghai Ninth People's Hospital and Jinshan Hospital between January 2018 and December 2022. Eligibility criteria for study inclusion were as follows: (a.) age ranging from 18 to 90 years; (b.) AIS diagnosis by cranial computed tomography (CT) and CT perfusion; (c.) presence of anterior circulation stroke secondary to large vessel occlusion (LVO); (d.) availability of complete clinical and laboratory data at admission. Patients were excluded if they met any of the following criteria: (a.) incomplete clinical or laboratory information; (b.) missing prognostic data; (c.) concurrent malignancy, or severe hepatic or renal dysfunction (defined as Child–Pugh class C or estimated glomerular filtration rate < 30 mL/min/1.73 m^2^).

The study protocol was approved by the Ethics Committee of the Ninth People's Hospital Affiliated with Shanghai Jiao Tong University School of Medicine (approval number: SH9H-2024-T417-1) and the Ethics Committee of Shanghai Jinshan Hospital (approval number: jszxyy202234). Given the retrospective nature of the study, the requirement for obtaining written informed consent from participants was waived. All procedures were conducted in strict adherence to the ethical principles outlined in the Declaration of Helsinki.

### Data collection

Baseline demographic characteristics, clinical variables, and laboratory parameters were extracted from electronic medical records upon patient admission. Demographic data included age, sex, smoking history, and alcoholism history. Vascular risk factors assessed included a history of hypertension, diabetes mellitus, hyperlipidemia, and previous stroke or transient ischemic attack (TIA).

Clinical features documented included collateral circulation status, which was evaluated using the hypoperfusion intensity ratio (HIR); poor collateral circulation was defined as HIR ≥ 0.4. Additional clinical variables included the time interval from symptom onset to randomization, baseline systolic blood pressure, baseline diastolic blood pressure, and baseline National Institutes of Health Stroke Scale (NIHSS) score. Stroke etiology was categorized according to the Trial of Org 10172 in Acute Stroke Treatment (TOAST) criteria into five subtypes: large-artery atherosclerosis, cardioembolism, small-vessel occlusion, stroke of other determined pathogenesis, and stroke of undetermined pathogenesis. The time interval from symptom onset to arterial puncture (OTP) was recorded as a key time-related variable.

Laboratory measurements were obtained within 24 h of admission, including fasting blood glucose concentration, leukocyte count, neutrophil count, and lymphocyte count. Derived inflammatory and coagulation-related indices included the neutrophil-to-lymphocyte ratio (NLR) and fibrinogen-to-albumin ratio (FAR). A composite inflammatory-coagulation index, designated as the NLR–FAR Index, was defined as the product of NLR and FAR. The multiplicative model was chosen based on the biological premise that systemic inflammation and coagulation/nutritional imbalance are not merely additive but likely interact synergistically within the thrombo-inflammatory cascade following stroke. This interaction term is hypothesized to better capture the compounded risk when both pathways are simultaneously activated, compared to an additive model.

Functional outcome was assessed at 30 days post-stroke using the modified Rankin Scale (mRS). A poor functional outcome was defined as mRS ≥ 3, while a favorable functional outcome was defined as mRS < 3. Follow-up assessments were conducted via outpatient clinic visits or structured telephone interviews by trained neurologists who were blinded to patients' imaging findings and laboratory results to minimize assessment bias.

### Statistical analysis

Continuous variables are presented as mean ± standard deviation (SD) for normally distributed data or median with interquartile range (IQR) for non-normally distributed data; categorical variables are expressed as counts and percentages. Between-group comparisons of continuous variables were performed using the Student's t-test (for normally distributed data) or the Mann–Whitney U-test (for non-normally distributed data). Categorical variables were compared using the Chi-square test or Fisher's exact test, as appropriate.

Univariable logistic regression analyses were performed to identify variables associated with poor functional outcome at 30 days. Variables with a *P*-value < 0.10 in univariable analyses, along with clinically relevant variables deemed important *a priori* based on existing literature (e.g., age, sex, smoking history, and alcoholism history), were considered for inclusion in the multivariable model. Before finalizing the multivariable model, we assessed multicollinearity among all candidate independent variables using the variance inflation factor (VIF). The final multivariable logistic regression models included the NLR Index, FAR Index and NLR-FAR Index exclusively as the primary predictors, adjusted for: age, sex, hypertension, smoking history, alcoholism history, admission systolic blood pressure, admission blood glucose, baseline NIHSS score, and OTP. Odds ratios (ORs) and their corresponding 95% confidence intervals (CIs) were reported to quantify the strength of associations. Restricted cubic spline (RCS) regression was employed to explore potential non-linear relationships between NLR–FAR Index and 30-day functional outcome; knots for RCS were placed at the 5th, 35th, 65th, and 95th percentiles of NLR–FAR Index distribution. Model fit and the presence of non-linearity were evaluated using Wald chi-square tests.

To assess the incremental predictive value of the NLR–FAR Index beyond conventional prognostic indicators, we performed the following analyses: 1. We constructed nested logistic regression models: Model 1 (Base model): Conventional clinical predictors only (age, sex, hypertension history, baseline NIHSS score, admission blood glucose, and onset-to-puncture time); Model 2 (Full model): Conventional predictors plus the NLR–FAR Index. Model performance was compared using the area under the receiver operating characteristic curve (AUC), Akaike Information Criterion (AIC), and likelihood-ratio tests. 2. Clinical utility assessment: Decision curve analysis was performed to evaluate the net benefit of incorporating the NLR–FAR Index into clinical decision-making across a range of threshold probabilities (1%−60%). This analysis compares the clinical utility of different strategies: treating all patients, treating no patients, and using model-based decisions. 3. We performed Hosmer-Lemeshow tests. A non-significant *P*-value (*P* > 0.05) indicates good calibration, meaning the model's predictions accurately reflect the actual event rates.

All statistical analyses were performed using R software (version 4.2.2; R Foundation for Statistical Computing, Vienna, Austria). A two-tailed *P*-value < 0.05 was considered statistically significant.

### Subgroup and interaction analyses

To evaluate the consistency of the NLR–FAR Index across different patient populations, we performed pre-specified subgroup analyses based on clinically relevant factors. Subgroups were defined as follows: age (< 65 vs. 65–80 vs. >80 years), sex (male vs. female), history of hypertension (yes vs. no), baseline NIHSS score (< 14 vs. ≥14), OTP (< 6 h vs. 6–12 h vs. 12–24 h vs. >24 h). Within each subgroup, multivariable logistic regression models were fitted with adjustment for potential confounders including sex (except in sex subgroup), hypertension history (except in hypertension subgroup), age (except in age subgroup), smoking history, drinking history, systolic blood pressure, blood glucose, baseline NIHSS score, and OTP. To formally test for effect modification, interaction terms between the NLR–FAR Index (as a continuous variable) and each subgroup variable were added to the full model, and significance was assessed using likelihood-ratio tests.

### Sensitivity analyses

To evaluate the robustness of our findings, we performed comprehensive sensitivity analyses. All the results of sensitivity analyses are presented in the Supplementary materials and consistently demonstrated significant associations between the NLR-FAR Index and poor functional outcomes. Extreme values are defined as being greater or less than 10 times the median. Sensitivity analysis includes excluding extreme values, adjusting only for gender and age, adjusting for all confounding factors, and comparing different outcome classifications. To rigorously assess the stability and validity of the identified NLR-FAR Index threshold, we performed internal validation using Bootstrap validation (500 iterations).

## Results

### Baseline characteristics

A total of 254 patients with acute ischemic stroke who underwent endovascular thrombectomy were included in this study, of whom 133 (52.4%) achieved favorable outcomes (mRS < 3) and 121 (47.6%) had unfavorable outcomes (mRS ≥3) at 30 days. The baseline demographic and clinical characteristics stratified by outcome status are summarized in Table 1.

**Table 1:** Baseline characteristics among patients with ischemic stroke undergoing endovascular treatment.

**Characteristics**	**Favorable outcome**	**Poor outcome**	***P* value**
*N*, number of patients	133	121	–
**Demographic features**			
Female	42 (31.6)	53 (43.8)	0.052
Age, y	68.8 ± 11.9	70.7 ± 12.7	0.233
Smoking history	18 (13.5)	28 (23.1)	0.068
Alcoholism history	13 (9.8)	19 (15.7)	0.218
**Medical history**			
Hypertension	75 (56.4)	96 (79.3)	<0.001
Diabetes mellitus	28 (21.1)	39 (32.2)	0.061
Hyperlipidemia	26 (19.5)	4 (3.3)	<0.001
Previous history of stroke or TIA	21 (15.8)	24 (19.8)	0.497
**Clinical features**			
Poor collateral circulation (HIR ≥ 0.4)	128 (96.2)	117 (96.7)	1.000
Time from onset to puncture, h	4 (2–6.7)	7.4 (4.6–11.8)	<0.001
Systolic BP, mmHg	140.1 ± 23.8	150.9 ± 22.5	<0.001
Diastolic BP, mmHg	78.8 ± 14.3	82.7 ± 12.6	0.022
Blood glucose, mmol/L	7.3 (6–9.6)	7.8 (6–10.8)	0.082
Baseline NIHSS score	9 (4-16)	15 (10-22)	<0.001
Ischemic stroke subtype	–	–	0.167
Large-artery atherosclerosis	45 (33.8)	28 (23.1)	–
Cardioembolism	47 (35.3)	51 (42.1)	–
Stroke of other determined pathogenesis	0 (0.0)	0 (0.0)	–
Stroke of undetermined pathogenesis	41 (30.8)	42 (34.7)	–

As shown in Table 1, patients with unfavorable outcomes (mRS ≥3) were older (70.7 ± 12.7 vs. 68.8 ± 11.9 years, *P* = 0.233), had higher prevalence of hypertension (79.3% vs. 56.4%, *P* < 0.001) and lower prevalence of hyperlipidemia (3.3% vs. 19.5%, *P* < 0.001), higher baseline NIHSS scores (median [IQR]: 15 [10–22] vs. 9 [4–16], *P* < 0.001), higher systolic blood pressure (150.9 ± 22.5 vs. 140.1 ± 23.8 mmHg, P < 0.001), higher diastolic blood pressure (82.7 ± 12.6 vs. 78.8 ± 14.3 mmHg, *P* = 0.022), longer OTP (median [IQR]: 7.4 [4.6–11.8] vs. 4 [2–6.7] h, *P* < 0.001), and tended to have higher blood glucose levels (median [IQR]: 7.8 [6–10.8] vs. 7.3 [6–9.6] mmol/L, *P* = 0.082). Additionally, there was a trend toward more females (43.8% vs. 31.6%, *P* = 0.052), higher smoking rates (23.1% vs. 13.5%, *P* = 0.068), and higher diabetes prevalence (32.2% vs. 21.1%, *P* = 0.061) in the unfavorable outcome group, although these differences did not reach statistical significance. These variables are well-established prognostic factors in ischemic stroke and may confound the association between inflammatory markers and outcomes. To account for these influences, we adjusted for these variables in multivariate logistic regression models.

We further examined Spearman correlations between key clinical variables and the three inflammatory-coagulation indices. Baseline NIHSS score showed significant positive correlations with NLR (ρ = 0.333, *P* < 0.001) and NLR-FAR Index (ρ = 0.354, *P* < 0.001), confirming that stroke severity is associated with heightened systemic inflammation. In contrast, GCS score was inversely correlated with NLR (ρ = −0.235, *P* = 0.0078) and NLR-FAR Index (ρ = −0.283, *P* = 0.0013), reflecting a link between impaired consciousness and inflammatory activation. Age showed positive correlations with NLR (ρ = 0.206, *P* = 0.0204) and NLR-FAR Index (ρ = 0.226, *P* = 0.0106). Hypertension was associated with higher NLR-FAR Index (ρ = 0.208, *P* = 0.0189). These correlations underscore the importance of adjusting for these confounders to isolate the independent prognostic value of the NLR-FAR Index.

Importantly, markers of systemic inflammation and coagulation-nutritional status differed substantially: the unfavorable group had higher median NLR (5.8 [3.2–8.9] vs. 3.1 [1.8–4.5], *P* < 0.001), FAR (0.08 [0.06–0.11] vs. 0.05 [0.04–0.07], *P* < 0.001), and the composite NLR-FAR Index (0.62 [0.38–0.91] vs. 0.25 [0.15–0.39], *P* < 0.001), establishing the rationale for further prognostic analysis.

### Association between NLR–FAR index and outcomes

#### Quintile-based analysis of the NLR–FAR index

**Figure 1:**
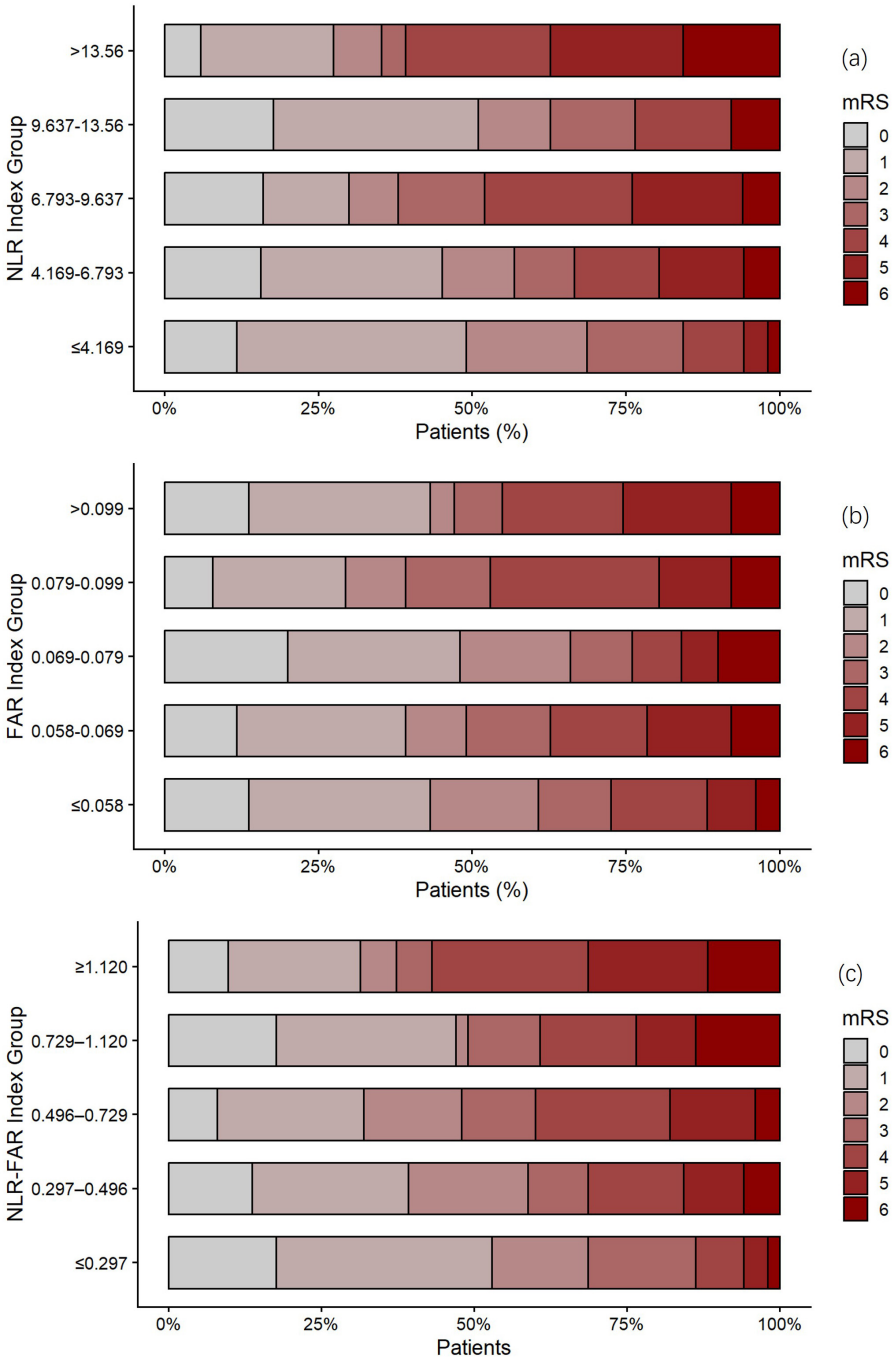
Stacked bar plots of 30-day modified Rankin Scale (mRS) scores according to quintiles of the: (a) NLR Index; (b) FAR Index; (c) NLR–FAR Index: Y-axis represents the percentage of patients in each mRS category within each quintile of the respective index.

To explore the gradient relationship between the NLR–FAR Index and outcomes, patients were stratified into quintiles (Q1: ≤ 0.297, Q2: 0.297–0.496, Q3: 0.496–0.729, Q4: 0.729–1.120, Q5: ≥1.120), with each group containing approximately 20% of the cohort. The distribution of mRS scores across quintiles (Figure 1) revealed a stepwise increase in unfavorable outcomes. Multivariable-adjusted analysis showed a clear dose-response relationship, with adjusted odds ratios increasing across quintiles: Q2 (*aOR* = 0.803, 95% CI: 0.293–2.200), Q3 (*aOR* = 1.621, 95% CI: 0.603–4.359), Q4 (*aOR* = 1.261, 95% CI: 0.476–3.341), and Q5 (*aOR* = 3.408, 95% CI: 1.194–9.728), with a significant trend *P*-value of 0.022. These data indicate that higher NLR–FAR Index values consistently predict worse functional recovery.

#### Logistic regression analysis

Multivariable logistic regression, adjusted for age, sex, hypertension, smoking history, alcoholism history, admission SBP, glucose, baseline NIHSS, and OTP (with no significant multicollinearity detected among these covariates, all VIFs < 2), quantified the independent predictive value of NLR, FAR, and the composite index.

Analysis by quintiles revealed distinct patterns for each index:

For the NLR Index, patients in the highest quintile (≥13.560) had a 3.054-folds higher risk of unfavorable outcomes (95% CI: 1.103–8.453, *P* = 0.032 for trend) compared to the lowest quintile (≤4.169), while other quintiles showed non-significant associations.

For the FAR Index, the highest quintile (≥0.099) showed a 1.519-folds increased risk (95% CI: 0.609–3.788), but no significant trend was observed across quintiles (*P* for trend = 0.370). Only the fourth quintile (0.079–0.099) showed a notable but non-significant elevation in risk (*aOR* = 1.849, 95% CI: 0.732–4.675).

For the composite NLR–FAR Index, patients in the highest quintile (≥1.120) had a 3.408-folds higher risk of unfavorable outcomes (95% CI: 1.194–9.728, *P* = 0.022 for trend) compared to the lowest quintile (≤0.297), demonstrating the strongest and most consistent dose-response relationship among the three indices. The trend test remained significant after full adjustment for confounders, confirming the robust predictive value of the NLR–FAR Index independent of established clinical variables.

#### Comparative predictive performance

The discriminative ability of each marker was assessed via logistic regression analysis. In univariate analysis, the NLR-FAR Index (*OR* = 1.964, 95% CI: 1.280–3.176, *P* = 0.004) demonstrated the strongest association with poor prognosis, so as NLR (*OR* = 1.067), while FAR alone showed no significance. After adjusting for clinical confounders, the NLR-FAR Index maintained significant association with adverse outcomes (adjusted *OR* = 1.910, 95% CI: 1.158–3.509, *P* = 0.026), as did NLR (adjusted *OR* = 1.073). These findings highlight that the combined NLR-FAR Index provide independent prognostic information after accounting for key clinical variables.

#### Restricted cubic spline analysis

**Figure 2:**
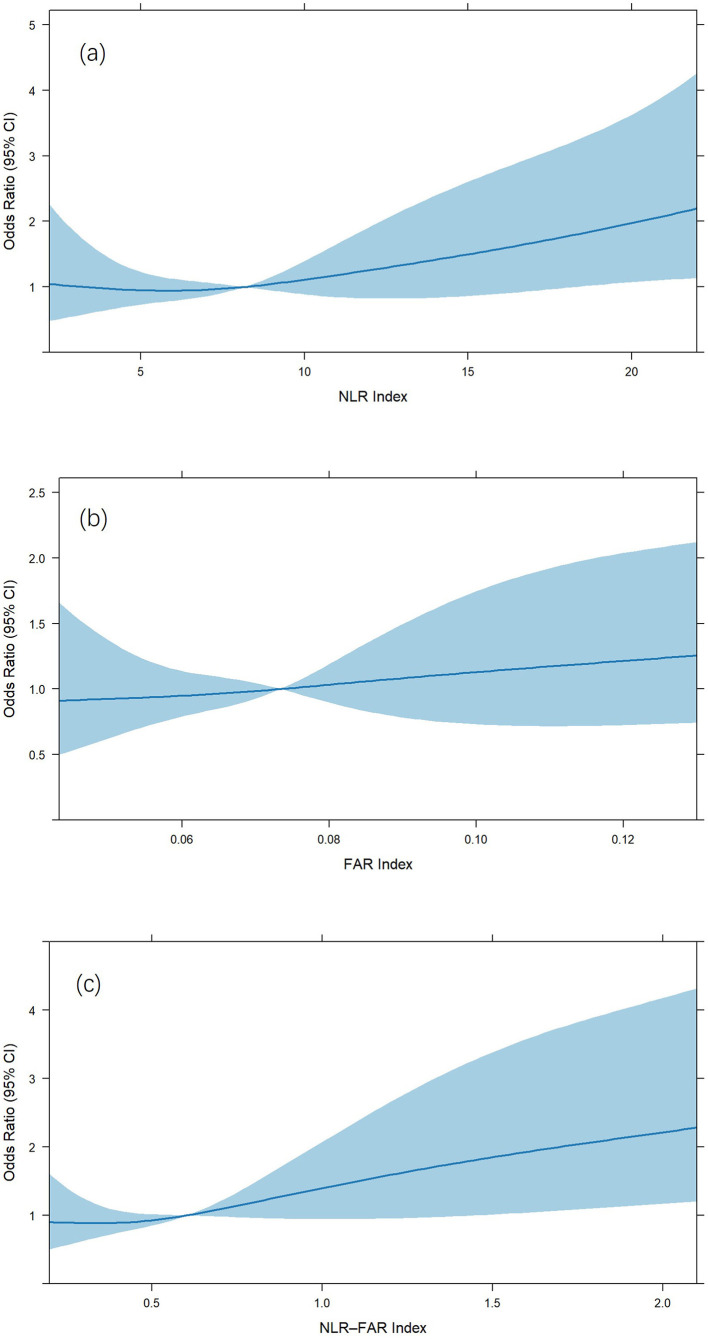
Restricted cubic spline analysis of the association between the: **(a)** NLR Index; **(b)** FAR Index; **(c)** NLR–FAR Index and risk of unfavorable outcome.

Restricted cubic spline analysis was performed to examine the association between the NLR–FAR Index and 30-day outcomes (Figure 2). Although no significant nonlinear relationship was detected (nonlinear *P* = 0.658), the index demonstrated a significant linear association with 30-day outcomes (overall *P* = 0.017). Importantly, the risk of unfavorable 30-day outcomes began to increase notably when the NLR–FAR Index exceeded a threshold value of 0.62, with odds ratios (ORs) consistently surpassing 1.0 beyond this point. This finding indicates that 0.62 may serve as a clinically meaningful cutoff for elevated risk. In the multivariate regression model adjusted for confounding factors, the NLR–FAR Index remained an independent and significant predictor of 30-day outcomes (*P* = 0.017), along with gender (*P* = 0.001), smoking history (*P* = 0.013), admission systolic blood pressure (*P* = 0.009), admission blood glucose (*P* = 0.022), admission NIHSS score (*P* < 0.001), and OTP (*P* = 0.0002). The discriminative ability of the model was good, with a C–index of 0.731, reinforcing the robustness of the linear association between the NLR–FAR Index and functional outcomes at 30 days.

#### Incremental Value of the NLR–FAR Index Beyond Conventional Predictors

**Figure 3:**
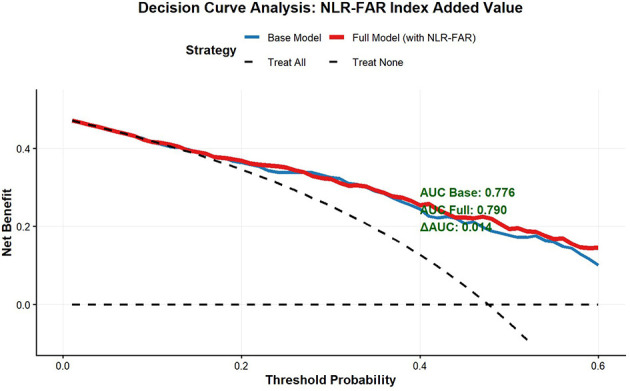
Decision curve analysis comparing the net benefit of models with and without the NLR–FAR index for predicting poor 30-day functional outcome after endovascular thrombectomy.

The addition of the NLR–FAR Index to conventional clinical predictors significantly improved prognostic accuracy (Table 2). The AUC increased from 0.790 for the baseline model to 0.880 for the full model, representing an absolute improvement of Δ*AUC* = 0.090 (95% CI: 0.023–0.155, *P* = 0.014). Decision curve analysis demonstrated that the model incorporating the NLR–FAR Index provided superior net benefit compared to conventional predictors alone across a clinically relevant range of threshold probabilities (Figure 3). The calibration performance of our prediction models was assessed using Hosmer-Lemeshow tests. The full model yielded a Hosmer-Lemeshow χ^2^ statistic of 6.375 (*P* = 0.783), while the baseline model showed a χ^2^ of 11.140 (*P* = 0.347). These non-significant *P*-values (*P* > 0.05) indicate that we cannot reject the null hypothesis of good fit. Bootstrap resampling with 500 iterations yielded an AUC of 0.742 (95% CI: 0.671–0.838).

**Table 2:** Improvement in predictive performance with NLR-FAR index addition.

**Model**	**AUC (95% CI)**	***P*-value**
Base (Conventional only^*^)	0.790 (0.776–0.790)	–
ΔAUC	0.090 (0.023–0.155)	0.014
Net Reclassification	0.124 (0.066–0.187)	<0.001

^*^Conventional predictors: age, sex, hypertension history, baseline NIHSS score, admission blood glucose, OTP.

#### Subgroup analyses of the NLR–FAR index

Subgroup analyses were conducted to evaluate the consistency of the NLR–FAR Index across different patient populations (Table 3). The predictive performance of the NLR–FAR Index showed variation across subgroups, with statistically significant interactions identified for OTP. Notably, the strongest association was observed in patients treated 6–12 h after symptom onset (P < 0.001). In contrast, no significant associations were found for patients treated within 6 h (*P* = 0.586) or between 12 and 24 h (*P* = 0.578). A significant association was also observed in patients aged >80 years (adjusted *OR* = 5.376, 95% CI: 1.008–28.663, *P* = 0.049), while no significant associations were found in younger age groups, with no significant overall age interaction. No significant associations were found in both males (*P* = 0.109) and females (*P* = 0.115). Similarly, no significant associations were observed in both non-hypertensive (*P* = 0.119) and hypertensive patients (*P* = 0.152). Regarding stroke severity, a trend toward stronger association was observed in patients with NIHSS < 14 (*P* = 0.053) compared to NIHSS ≥14 (*P* = 0.527).

**Table 3:** Subgroup analyses of the NLR–FAR Index for predicting poor functional outcome.

**Subgroup**		***N***	**Poor outcome, *n* (%)**	**Adjusted OR (95% CI)**	***P*-value**	***P* for interaction**
Age	< 65 years	81	40 (49.4)	1.792 (0.578–5.550)	0.312	0.242
	65–80 years	123	51 (41.5)	1.315 (0.663–2.606)	0.434	
	>80 years	50	30 (60.0)	5.376 (1.008–28.663)	0.049^*^	
Sex	Male	159	68 (42.8)	1.785 (0.878–3.626)	0.109	0.455
	Female	95	53 (55.8)	2.428 (0.806–7.314)	0.115	
Hypertension history	No	83	25 (30.1)	2.816 (0.765–10.361)	0.119	0.326
	Yes	171	96 (56.1)	1.599 (0.842–3.037)	0.152	
Baseline NIHSS score	< 14	143	54 (37.8)	2.937 (0.988–5.813)	0.053	0.095
	≥14	111	67 (60.4)	1.236 (0.641–2.382)	0.527	
OTP	< 6 h	126	41(32.5)	1.195 (0.630–2.267)	0.586	0.010^*^
	6–12 h	81	50 (61.7)	16.245 (2.918–90.429)	<0.001	
	12–24 h	38	22 (57.9)	0.689 (0.186–2.557)	0.578	

^*^Adjusted for sex, hypertension history, age, smoking history, drinking history, systolic blood pressure, blood glucose, baseline NIHSS score, and OTP.

### Sensitivity analyses

Multiple sensitivity analyses confirmed the robustness of our primary findings (Supplementary Table S1). Excluding extreme NLR-FAR Index values maintained a significant association with unfavorable outcome (adjusted *OR* = 1.853, 95% CI: 1.045–3.286, *P* = 0.035). Different adjustment strategies yielded consistent results, from minimal adjustment (*OR* = 2.067, 95% CI: 1.276–3.350, *P* = 0.003) to full adjustment (*OR* = 1.910, 95% CI: 1.079–3.384, *P* = 0.026). When evaluating alternative outcome definitions, the NLR-FAR Index showed significant associations with both mRS ≥3 (*OR* = 1.910, *P* = 0.026) and mRS ≥4 (*OR* = 1.934, 95% CI: 1.125–3.324, *P* = 0.017), but not with the less stringent mRS ≥2 (*OR* = 1.397, 95% CI: 0.831–2.351, *P* = 0.207), suggesting greater predictive value for severe disability. Overall, effect estimates ranged consistently from 1.397 to 2.067 across all analyses.

## Discussion

In this study, we demonstrated that the NLR, FAR, and particularly the composite NLR–FAR Index were associated with 30-day functional outcomes in patients undergoing EVT for acute large-vessel occlusion stroke. Through quantitative analysis of their relationships and predictive performance, we further clarified the distinct clinical value of each marker: the NLR–FAR Index exhibited superior predictive power compared with NLR or FAR alone. These findings collectively suggest that the NLR–FAR Index may serve as a clinically practical and robust biomarker for early risk stratification, potentially guiding individualized management strategies (e.g., intensified monitoring, targeted anti-inflammatory or nutritional interventions) after EVT.

Our findings align with prior evidence that systemic inflammation and metabolic dysfunction are key drivers of stroke risk and prognosis. For example, an Estonian population-based study identified multiple routine laboratory markers—such as red cell distribution width, lymphocyte-to-neutrophil ratio, and renal function indices—as early predictors of ischemic stroke (21), highlighting the prognostic relevance of systemic physiological perturbations. Consistent with previous reports in EVT cohorts, our results confirmed that higher NLR values were significantly associated with poor 30-day outcomes (7, 8, 11–13). Although the NLR calculation is simpler, its risk association exhibits non-monotonic fluctuations (with the intermediate group having the highest risk), which may limit its stable application across different patient subgroups. The NLR-FAR index demonstrates a more consistent increasing trend and is more suitable as a continuous risk assessment tool.

Contrary to previous research findings (16, 18, 26), this study discovered that the FAR index alone is not effective in predicting 30-day functional prognosis after EVT. There is a lack of a significant dose-response relationship between the index and outcomes, and the risk estimates for all quintile groups were not statistically significant. Based on the results presented in Table 4, it is speculated that the risk pattern may exhibit a U-shaped or J-shaped relationship, which requires further research for verification.

**Table 4:** ORs and 95% CIs of 30-Day clinical outcomes (mRS score ≥ 3) according to the quintiles of NLR, FAR, and NLR–FAR index in ischemic stroke patients undergoing endovascular treatment.

**Variable**	**Patients, *n* (%)**	**Unadjusted OR (95%CI)**	**Multiple adjusted OR (95%CI)**
**NLR Index**			
≤ 4.169	51 (20.1)	1.000	1.000
4.169–6.793	51 (20.1)	1.659 (0.738–3.732)	1.132 (0.410–3.125)
6.793–9.637	50 (19.6)	3.569 (1.569–8.121)	2.424 (0.909–6.460)
9.637–113.560	51 (20.1)	1.299 (0.572–2.948)	1.215 (0.459–3.219)
≥13.560	51 (20.1)	4.010 (1.758–9.146)	3.054 (1.103–8.453)
*P*_trend_	–	<0.001	0.032
**FAR Index**			
≤0.058	51 (20.1)	1.000	1.000
0.058–0.069	51 (20.1)	1.612 (0.735–3.537)	1.191 (0.469–3.023)
0.069–0.079	50 (19.6)	0.798 (0.355–1.797)	0.611 (0.234–1.601)
0.079–0.099	51 (20.1)	2.402 (1.085–5.320)	1.849 (0.732–4.675)
≥0.099	51 (20.1)	1.744 (0.794–3.828)	1.519 (0.609–3.788)
*P*_trend_	–	0.166	0.370
**NLR–FAR Index**			
≤0.297	51 (20.1)	1.000	1.000
0.297–0.496	51 (20.1)	1.531 (0.679–3.452)	0.803 (0.293–2.200)
0.496–0.729	50 (19.6)	2.370 (1.053–5.332)	1.621 (0.603–4.359)
0.729–1.120	51 (20.1)	2.275 (1.015–5.099)	1.261 (0.476–3.341)
≥1.120	51 (20.1)	3.684 (1.623–8.363)	3.408 (1.194–9.728)
*P*_trend_	–	0.002	0.022

A major methodological innovation of this study is the integration of NLR and FAR into a composite NLR–FAR Index. Unlike previous studies that have typically assessed single inflammatory or coagulation markers in isolation (22–26), our approach reflects both systemic immune activation and coagulation dysfunction simultaneously. This integration is biologically meaningful, as LVO stroke pathogenesis and post-EVT recovery are shaped by the complex interplay between inflammation and thrombosis (27–30). Our data confirm that this holistic capture of pathophysiology translates to superior predictive performance: the NLR–FAR Index showed the strongest risk amplification and the lowest risk threshold among the three markers. Moreover, by employing multivariable logistic regression models adjusted for baseline demographics (age, sex), vascular risk factors (hypertension, smoking history, alcoholism history), and clinical severity (admission NIHSS, OTP), we demonstrated that the NLR–FAR Index's predictive ability remained robust.

Beyond confirming its overall prognostic utility, our study revealed a linear relationship between the NLR–FAR Index and poor 30-day outcomes. The restricted cubic spline analysis further elucidated this relationship, demonstrating that while the overall association was primarily linear (nonlinear *p* = 0.658), there was a clear inflection point around *NLR-FAR* = 0.62, beyond which the odds of poor outcome consistently exceeded 1.0. This reinforces the clinical utility of the 0.62 threshold identified in our decision curve analysis. To our knowledge, this is the first study to identify such a distinct threshold effect in an EVT population. RCS analysis revealed that the risk of poor outcome significantly increased at NLR-FAR values above 0.62, with odds ratios exceeding 1.0. This supports the use of 0.62 as a clinically meaningful threshold for risk stratification. Patients with NLR–FAR Index >0.62 at admission may benefit from targeted interventions, including intensified neurological monitoring and adjunctive anti-inflammatory therapy (31, 32).

Our study demonstrates that the NLR–FAR Index provides significant incremental value beyond established clinical prognostic indicators. This suggests that the index captures distinct aspects of the complex inflammatory and coagulative response to cerebral ischemia that are not fully reflected by conventional clinical measures. The improvement in discriminative ability supports the clinical utility of incorporating this biomarker into existing prognostic frameworks. Decision curve analysis further substantiates the clinical relevance of these findings, particularly in identifying patients who might benefit from more intensive monitoring, adjunctive anti-inflammatory therapies, or rehabilitation strategies. While the NIHSS is the gold standard for assessing initial stroke severity and the mRS for defining functional outcome, our study demonstrates that the NLR–FAR index, derived from routine blood tests, provides complementary prognostic information that is independent of and additive to these established clinical assessments. This suggests it captures a distinct pathophysiological domain (thrombo-inflammation) not fully reflected by traditional scales.

Subgroup analysis provides important insights into the differential expression of NLR-FAR index in patient populations. This index shows generally consistent predictive value at most clinical levels. It is worth noting that we observed that the NLR-FAR index may be particularly valuable in risk stratification at 6–12 h, as inflammation and coagulation processes may have the greatest impact on clinical outcomes. Age stratification analysis shows that patients over 80 years old have a stronger association trend, indicating that utility may be improved in the older adults population. There was no significant interaction effect between gender, history of hypertension, and baseline NIHSS score, which enhances the universality of this biomarker. The consistent performance of these key clinical dimensions suggests that regardless of these baseline features, the NLR-FAR index may be applicable to a wide range of AIS patients undergoing EVT.

We further evaluated the robustness of our findings through comprehensive sensitivity analysis (Supplementary Table S1). When changing the definition of adverse outcomes, the correlation between mRS ≥ 3 and mRS ≥ 4 remained statistically significant, consistent with our preliminary analysis. However, when using mRS ≥ 2 as the result, this association was not significant, indicating that the NLR-FAR index may be more specifically used to identify patients with more severe disabilities. Further analysis confirmed the stability we found: excluding extreme values produced similar results, as did the models with minimal and complete adjustments. Among various analytical methods, these consistent findings enhance people's confidence in the primary association between NLR-FAR index and adverse functional outcomes after EVT.

### Limitations

Several limitations should be acknowledged. First, this was a retrospective, single-center study with a relatively small sample size, which may restrict the generalizability of the findings. Validation through larger, multicenter prospective studies is warranted to verify the index's applicability in various regions and ethnic groups. Second, the exclusion of patients with incomplete follow-up data or severe organ dysfunction—while necessary to preserve data integrity and reduce confounding in this initial analysis—may limit applicability to broader stroke populations, particularly those with substantial comorbidities. Third, despite adjustment for multiple confounding variables, the possibility of residual confounding from unmeasured or imperfectly measured factors (such as detailed nutritional status, body mass index, or subtle metabolic parameters) cannot be entirely ruled out. Fourth, the NLR–FAR index was derived from a single blood sample obtained at admission, which may not accurately reflect dynamic changes in inflammatory status over time; serial measurements could provide deeper insights into the temporal relationship between systemic inflammation and stroke outcomes. Fifth, the biological mechanisms linking the NLR–FAR index to stroke prognosis remain speculative. Further exploration of the NLR–FAR index's combined effects with other biomarkers, including integration with multi-level biological data from genomics and metabolomics is required, and investigation of its application in long-term follow-up studies, especially in assessing post-stroke recovery outcomes and complication risks. Finally, the observed threshold of 0.62 was derived from restricted cubic spline and decision curve analyses. However, this cutoff should be considered a preliminary, data-driven reference value. Its generalizability, particularly across subgroups such as patients with different onset-to-puncture times where we observed significant effect modification, and its optimal calibration for clinical application require confirmation in larger, prospective, and multi-center cohorts.

## Conclusion

In summary, our study demonstrates that the composite NLR–FAR Index outperforms its individual components (NLR and FAR) in predicting 30-day functional outcomes after EVT for LVO stroke, with risk increasing at values above 0.62. Sensitivity analyses confirmed the robustness of this association for more severe disability. Subgroup analyses suggest its predictive value may be particularly pronounced in patients treated within 6–12 h of symptom onset. These findings highlight the NLR–FAR Index's potential as a simple, cost-effective biomarker for early risk stratification, especially in specific clinical windows. They provide a basis for future prospective trials exploring NLR–FAR Index-guided individualized management strategies to improve post-EVT recovery.

## Data Availability

The raw data supporting the conclusions of this article will be made available by the authors, without undue reservation.
