# Supplementary material for: NLR–FAR Index as a superior predictor of 30-day functional outcome after endovascular thrombectomy in acute ischemic stroke
Source: Front Neurol. 2026 Feb 25;17:1703841. doi: 10.3389/fneur.2026.1703841 (PMC12975429; doi:10.3389/fneur.2026.1703841)
Supplement: Supplementary file 1 [file Table_1.DOCX]

Table S1. Sensitivity analyses of the NLR-FAR Index association with poor functional outcome.

| Analysis Type | OR (95% CI) | P value |
| --- | --- | --- |
| Primary analysis | 1.910 (1.080-3.380) | 0.026 |
| excluding extreme values | 1.853 (1.045-3.286) | 0.035 |
| Minimal adjustment | 2.067 (1.276-3.350) | 0.003 |
| Full adjustment | 1.910 (1.079-3.384) | 0.026 |
| mRS ≥2 | 1.397 (0.831-2.351) | 0.207 |
| mRS ≥3 | 1.910 (1.079-3.384) | 0.026 |
| mRS ≥4 | 1.934 (1.125-3.324) | 0.017 |

Table S2. Model calibration assessment using Hosmer-Lemeshow goodness-of-fit test

| Model | χ² Statistic | df | P value |
| --- | --- | --- | --- |
| Baseline model (without NLR–FAR) | 11.140 | 10 | 0. 347 |
| Full model (with NLR–FAR) | 6.375 | 10 | 0.783 |
